# Mortality patterns among COVID‐19 patients in two Saudi hospitals: Demographics, etiology, and treatment

**DOI:** 10.1111/irv.13127

**Published:** 2023-03-21

**Authors:** Fatimah S. AlGhawi, Sami S. AlMudarra, Abdullah M. Assiri

**Affiliations:** ^1^ Field Epidemiology Training Program Ministry of Health Riyadh Saudi Arabia; ^2^ Public Health Ministry of Health Riyadh Saudi Arabia

**Keywords:** COVID‐19, Dammam, eastern, Medina, mortality, western

## Abstract

**Background:**

Saudi Arabia (SA) reported its first case of COVID‐19 on 2 March 2020. Mortality varied nationwide; by April 14, 2020, Medina had 16% of SA's total COVID‐19 cases and 40% of all COVID‐19 deaths. A team of epidemiologists investigated to identify factors impacting survival.

**Methods:**

We reviewed medical records from two hospitals: Hospital A in Medina and Hospital B in Dammam. All patients with a registered COVID‐related death between March and May 1, 2020, were included. We collected data on demographics, chronic health conditions, clinical presentation, and treatment. We analyzed data using SPSS.

**Results:**

We identified 76 cases: 38 cases from each hospital. More fatalities were among non‐Saudis at Hospital A (89%) versus Hospital B (82%, *p* < 0.001). Hypertension prevalence was higher among cases at Hospital B (42%) versus Hospital A (21%) (*p* < 0.05). We found statistically significant differences (*p* < 0.05) in symptoms at initial presentation among cases at Hospital B versus Hospital A, including body temperature (38°C vs. 37°C), heart rate (104 bpm vs. 89 bpm), and regular breathing rhythms (61% vs. 55%). Fewer cases (50%) at Hospital A received heparin versus Hospital B (97%, *p*‐value < 0.001).

**Conclusion:**

Patients who died typically presented with more severe illnesses and were more likely to have underlying health conditions. Migrant workers may be at increased risk due to poorer baseline health and reluctance to seek care. This highlights the importance of cross‐cultural outreach to prevent deaths. Health education efforts should be multilingual and accommodate all literacy levels.

## 1 INTRODUCTION

COVID‐19, a global pandemic that first appeared in China in December 2019, has quickly spread across the globe since the first case was reported.^1^ KSA had its first case on March 2, 2020; by March 23, 2022, case had been reported nationwide. On April 14, the Saudi Field Epidemiology Training Program (FETP) investigated a cluster of COVID‐19 deaths in Medina. The majority of deaths occurred in Hospital A, which was Medina's designated COVID‐19 hospital. At that time, Medina had 16% of KSA's total COVID‐19 cases and 40% of all COVID‐19 deaths. Subsequently, Hospital B in Dammam was chosen for comparison as it was Dammam's designated COVID‐19 hospital.

Clark et al.^2^ developed a prediction model that estimated a potential occurrence of 1.0 to 2.4 billion severe COVID‐19 infections among people with severe clinical conditions such as cardiovascular disease, chronic kidney disease, chronic respiratory disease, and diabetes.

The severity of the disease varies significantly, ranging from asymptomatic infection to the development of severe complications and death.^3^ Age, gender, and the presence of co‐morbidities have been reported to be contributing factors to COVID‐19 severity.^4^, ^5^, ^6^, ^7^ Patients with diabetes or chronic obstructive pulmonary disease (COPD) are more likely to have longer hospitalizations, be admitted to intensive care units (ICU), and require mechanical ventilation.^5^, ^8^ Conversely, mild prognosis has been reported among pediatric cases. However, the severity of the infection has also been reported among children affecting up to 5% of the infected patients although compared with adults, children and/or adolescents tend to have a mild COVID‐19 course with a good prognosis.^9^, ^10^, ^11^

Evidence on the pathology behind the development of the disease has also been variable. At first, it was thought that the virus affects the respiratory tract only; however, reports showed that it could affect many organs, including the blood, heart, brain, kidneys, pancreas, and eyes.^12^, ^13^ Moreover, the severity of the infection has also been related to several laboratory variables. Prothrombin time, C‐reactive protein, D‐dimer, procalcitonin, and fibrinogen levels have reportedly been associated with the deterioration of the disease.^14^, ^15^, ^16^, ^17^, ^18^ Some of these biomarkers have helped in building prediction models to decrease mortality among critically‐ill COVID‐19 patients.^19^, ^20^ Other investigations have reported an association between patients' blood type and the prognosis of the infection.^21^, ^22^ This indicates the fact that mortality because of COVID‐19 is different due to the different epidemiology among the affected populations.^23^ For that, we aim to assess and compare the different factors related to COVID‐19 mortality, including patients' demographics, clinical characteristics, and treatment regimens used among patients of two Saudi hospitals.

## 2 METHODS

### 2.1 Study design

We conducted a retrospective study by reviewing the patients' records at Hospital A in Medina and Hospital B in Dammam. The study was conducted between March 2020 to May 1, 2020.

### 2.2 Subject selection and procedure

All confirmed COVID‐19 patients with a registered COVID‐related death were included in the current study. No restrictions were made regarding age, gender, nationality, or the admitting department. We excluded deaths not certified as a direct result of COVID‐19 or patients with suspected COVID‐19 status.

Patients' demographics, documented deaths, cause of death, treatments provided, admission details, and hospital stay details were all collected. We reviewed records for cases between March 2020 and May 1, 2020.

### 2.3 Data analysis

Data entry and analyses were conducted using SPSS v.26 (IBM, NY). Nominal variables were presented as frequencies (n) and percentages (%). The Chi^2^ test (or Fisher's exact test, as appropriate) was used for identifying differences between hospitals. The continuous variables were presented as means and standard deviations (SDs). We used a t‐test or Mann–Whitney test based on the distribution of the data (normally distributed or not).

A binary logistic regression model was constructed to control any potential confounders and determine the significantly associated factors with the mortality outcome. The odds ratio and 95% confidence interval ([95% CI]) are presented. Statistical significance was set at a *P‐*value < 0.05 for all analyses.

### 2.4 Informed consent and ethical considerations

No identifying information on any patient was collected, and all collected data were exclusively used for statistical analysis. All data were kept confidential. Before commencement, the study protocol was cleared by the institutional review board and the ethics committee at King Fahad Medical City, Riyadh, Saudi Arabia.

## 3 RESULTS

### 3.1 Baseline characteristics

We identified 76 patients that met the inclusion criteria; 38 were patients at Hospital A, and 38 were patients at Hospital B. The mean age of the included patients was 51.7 years, and 93% of the patients were male. We found no statistically significant differences among the included patients between both hospitals in terms of age (*P‐*value = 0.322), gender (*P‐*value = 0.500) or the percentage of overweight (*P‐*value = 0.911). In contrast, we found a statically significant difference in the nationality distribution among the aforementioned two hospitals (*P‐*value < 0.001) (Table 1).

**TABLE 1:** Baseline characteristics of the included patients.

Variable		Hospital B		Hospital A		Total		*P*‐value
		*n*	%	*n*	%	*N*	%	
Age; Mean ± SD		53.1 ± 11.2		50.3 ± 13.3		51.7 ± 12.3		0.322
Gender	Female	3	7.9	2	5.3	5	6.6	0.500
	Male	35	92.1	36	94.7	71	93.4	
BMI > 25 kg/m^2^	No	16	42.1	7	43.8	23	42.6	0.911
	Yes	22	57.9	9	56.3	31	57.4	
Healthcare worker	No	37	97.4	36	94.7	73	96.1	0.556
	Yes	1	2.6	2	5.3	3	3.9	
Nationality	Saudi	7	18.4	4	10.5	11	14.5	<0.001*
	Non‐Saudi	31	81.6	31	89.5	65	85.5	
Smoker	Yes	3	7.9	6	15.8	9	11.8	0.578
	No	21	55.3	21	55.3	42	55.3	
	Unknown	14	36.8	11	28.9	25	32.9	
Comorbidities	No	18	47.4	22	57.9	40	52.6	0.358
	Yes	20	52.6	16	42.1	36	47.4	
Diabetes mellitus	Yes	13	34.2	14	36.8	27	35.5	0.811
	No	25	65.7	24	63.1	49	64.4	
Hypertension	Yes	16	42.1	8	21.1	24	31.6	0.048*
	No	22	57.8	30	78.9	52	68.4	
Ischemic deart disease	Yes	6	15.8	2	5.3	8	10.5	0.135
	No	32	84.2	36	94.7	68	89.4	

Abbreviations: SD, standard deviation; BMI, body mass index.

* Statistically significant.

Only 12% of the patients reported smoking; 47% of the patients had one or more documented comorbidities. The most prevalent comorbidity was diabetes mellitus (DM), being present in 36% of the patients, followed by hypertension (32%) and ischemic heart disease (IHD) in 11%. We also found a statistically significant difference in the prevalence rates of hypertension between patients between the two hospitals (*P*‐value = 0.048) (Table 1).

### 3.2 Patients' admission details and baseline clinical data

The mean admission body temperature of all patients was 37.7°C, whereas their mean initial respiratory rate was 26.6 breaths per minute, and the mean initial heart rate was 98.6 ± 19.3 beats per minute. The mean visual triage score was 6.7 ± 1.9, and the mean Glasgow Coma Scale was 10.2 ± 5.6. For Hospital B, the mean visual triage score was 6.3 ± 2.5, while the mean Glasgow Coma Scale was 10.8 ± 5.4. For those patients with available data, 76% of the patients were registered as an emergency, 17% were directly admitted to the ICU, and 7% were registered from the outpatient department. In the same context, the admission source was variable among the included patients; 30.3% of the patients were admitted from the emergency room, 26% were referred from another hospital, 22% were admitted from the clinic, and 21.1% were in the hospital ward. Most of the patients (76%) did not sign a “Do not resuscitate” form, while only 24% did sign it. Nevertheless, there was a statistically significant difference between Hospital B and Hospital A in terms of initial body temperature (*P‐*value = 0.001), initial heart rate (*P‐*value = 0.002), and registry type (*P*‐value< 0001) (Tables 2 and 3).

**TABLE 2:** Patients' baseline clinical data.

Variables	Hospital B		Hospital A		Total		*P*‐value
	Mean	SD	Mean	SD	Mean	SD	
Initial temperature	37.9 ± 1.0		37.4 ± 0.7		37.7 ± 0.9		0.011*
Initial respiratory rate	25.6 ± 10.2		27.8 ± 10.4		26.6 ± 10.3		0.392
Initial heart rate (bpm)	104 ± 16.9		88.9 ± 19.4		98.6 ± 19.3		0.002*
Visual triage score	6.3 ± 2.5		7.1 ± 0.8		6.7 ± 1.9		0.293
Glasgow Coma Scale	10.8 ± 5.4		9.5 ± 5.8		10.2 ± 5.6		0.386

Abbreviation: SD, standard deviation.

* Statistically significant.

**TABLE 3:** Patients' registry type.

Variable	Hospital B		Hospital A		Total		*P*‐value
	*n*	%	*n*	%	*N*	%	
Direct ICU admission	13	34.2	0	0	13	17.1	<0.0001*
Emergency	25	65.8	33	86.8	58	76.3	
OPD	0	0	5	13.2	5	6.6	

* Statistically significant.

On admission, 79% of the included patients presented with fever, 76% with shortness of breath, 10% with a sore throat, and 75% with a cough. Regarding the cough type at presentation, 16% of the included patients presented with productive cough, and 15% presented with non‐productive cough, whereas the remaining portion either did not have a cough or did not have a documented cough type. We did not find any statistically significant differences regarding fever (*P‐*value = 1.000), shortness of breath (*P‐*value = 1.000), sore throat (*P‐*value = 0.711), cough (*P‐*value = 0.791), or type of cough among the included patients (*P‐*value = 0.250) among the included patients (Table 4).

**TABLE 4:** General clinical signs and symptoms at the presentation.

Variables		Hospital B		Hospital A		Total		*P*‐value
		*n*	%	*n*	%	*N*	%	
Fever	No	8	21.1	8	21.1	16	21.1	1.000
	Yes	30	78.9	30	78.9	60	78.9	
Shortness of breath	No	9	23.7	9	23.7	18	23.7	1.000
	Yes	29	76.3	29	76.3	58	76.3	
Sore throat	No	35	92.1	33	86.8	68	89.5	0.711
	Yes	3	7.9	5	13.2	8	10.5	
Cough	No	10	26.3	9	23.7	19	25.0	0.791
	Yes	28	73.7	29	76.3	57	75.0	
Cough type	Non‐productive	8	21.1	3	7.9	11	14.5	0.250
	Productive	5	13.2	7	18.4	12	15.8	
	None/NA	25	65.8	28	73.7	53	69.7	
Breathing rhythm	Regular	23	60.5	21	55.3	44	57.9	0.017*
	Irregular	1	2.6	9	23.7	10	13.2	
	Not available	14	36.8	8	21.1	22	28.9	
Breathing quality	Normal	23	60.5	21	55.5	44	57.9	0.029*
	Labored	1	2.6	7	18.4	8	10.5	
	Not available	14	36.8	10	26.3	24	31.1	

* Statistically significant.

### 3.3 Comparison of patients' findings and examination results

The mean O_2_ saturation at the admission was 83.9 ± 9.2, with a mean of 85.4 ± 8.5 and 82.5 ± 9.8 at Hospital B and Hospital A, respectively. Regarding the radiological findings, bilateral infiltrates were present in 29% of the included patients at admission, with a prevalence of 26% at Hospital B and 32% in Hospital A (Table 5).

**TABLE 5:** Radiological findings and O_2_ saturation on admission.

Variables		Hospital B		Hospital A		Total		*P*‐value
		*N*	%	*N*	%	*N*	%	
Initial O_2_ saturation (%); Mean ± SD		85.4 ± 8.5		82.5 ± 9.8		83.9 ± 9.2		0.173
Bilateral infiltrates	No	28	73.7	26	68.4	54	71.1	0.613
	Yes	10	26.3	12	31.6	22	28.9	

The local chest examination findings were variable among patients. The examination is usually carried out and documented by the attending physician according to the standard definitions during the initial assessment. Breathing rhythm was regular in 58% of the patients, irregular in 13% of the patients, and the data about the remaining 29% were not documented. For the breathing depth, 53% of the patients showed a normal depth of respiration, 10% showed shallow breathing, 7% showed deep breathing, and 30% did have a documented breathing depth. For the breathing quality, 57.9% of the patients showed a normal breathing quality, 10% showed labored breathing, and the remaining 32% did have a documented breathing depth. In the same context, 65% of the included patients did not have any added sounds; however, there was a high variability of the breathing added sounds among the remaining ones. Bilateral crepitations were found in 17% of the patients. Eight percent had wheezes, 7% had bilateral crackles, 3% had rhonchi, and 1% had scattered crepitations. There was a statistically significant difference between the two hospitals in the patterns of breathing rhythm (*P*‐value = 0.017), breathing quality (*P*‐value = 0.029), and added sounds (*P*‐value = 0.029) among the included patients (Tables 4 and 5).

### 3.4 Comparison of interventions/treatments used for patients in both hospitals

The majority of the patients (93%) were admitted to the ICU at some point, and most of the patients (89%) required ventilation during their treatment course. There was no statistically significant difference between hospitals in the ICU admission (*P*‐value = 0.644) or ventilation rates (*P*‐value = 0.262) among the included patients. Regarding drugs administered, about two‐thirds (64%) of the patients were treated with hydroxychloroquine, and most patients (74%) were treated with heparin. There was a statistically significant difference between the two hospitals in heparin usage rates (*P*‐value < 0.001), whereas hydroxychloroquine usage rates were comparable (*P*‐value = 0.811) (Table 6).

**TABLE 6:** Comparison of treatments used for patients in both hospitals.

Variables		Hospital B		Hospital A		Total		*P*‐value
		*n*	%	*n*	%	*N*	%	
ICU admission	No	3	7.9	2	5.3	5	6.6	0.644
	Yes	35	92.1	36	94.7	71	93.4	
Ventilated	No	2	5.3	6	15.8	8	10.5	0.262
	Yes	36	94.7	32	84.2	68	89.5	
Hydroxychloroquine use	No	14	36.8	13	34.2	27	35.5	0.811
	Yes	24	63.2	25	65.8	49	64.5	
Heparin use	No	1	2.6	19	50.0	20	26.3	<0.001*
	Yes	37	97.4	19	50.0	56	73.7	
Continuous renal replacement therapy	No	34	89.5	32	84.2	66	86.8	0.736
	Yes	4	10.5	6	15.8	10	13.2	

* Statistically significant.

### 3.5 Comparison of patients' outcomes

The mean hospital time of all included patients was 6.4 ± 4.5 days, with a mean of 7.1 ± 4.3 days and 5.6 ± 4.7 days in Hospital B and Hospital A, respectively. For the time span from admission to ICU admission, the meantime in days was 0.8 ± 1.4 days, with a mean of 0.9 ± 1.2 days and 0.8 ± 1.6 days in Hospital B and Hospital A, respectively. For the time span from admission to ventilation, the meantime in days was 1.6 ± 2.3 days, with a mean of 1.6 ± 2.3 days and 1.5 ± 2.2 days in Hospital B and Hospital A, respectively. For the time span from ICU admission to death, the meantime in days was 5.6 ± 4.3 days, with a mean of 6.2 ± 4.5 days and 5.1 ± 4.1 days in Hospital B and Hospital A, respectively. For the time span from ventilation to death, the meantime in days was 5.5 ± 4.3 days, with a mean of 6.0 ± 4.3 days and 4.9 ± 4.3 days in Hospital B and Hospital A, respectively. There were no statistically significant differences between the two hospitals in all recorded outcomes (Table 7).

**TABLE 7:** Comparison of different patient outcomes between the two hospitals.

Variables	Hospital B		Hospital A		Total		*P*‐value
	Mean	SD	Mean	SD	Mean	SD	
Hospital time (Days)	7.1	4.3	5.6	4.7	6.4	4.5	0.158
Time to ICU (Days)	0.9	1.2	0.8	1.6	0.8	1.4	0.753
Time to vent (Days)	1.6	2.3	1.5	2.2	1.6	2.3	0.945
ICU to death time (Days)	6.2	4.5	5.1	4.1	5.6	4.3	0.263
Ventilation to death (Days)	6.0	4.3	4.9	4.3	5.5	4.3	0.310

Abbreviation: SD, standard deviation.

### 3.6 Effect of hospital choice on patients' outcomes

Logistic regression was performed to test whether the hospital choice has an effect on the patients' outcomes or not. This was tested using Hospital A as a reference and doing the test for choosing Hospital B. Accordingly, there was a reduction in all outcomes in Hospital B when compared with Hospital A. Length of hospitalization (OR = 0.93; 95% CI = 0.83–1.03), time from admission to ICU (OR = 0.95; 95% CI = 0.68–1.32), time from admission to ventilation (OR = 0.99; 95% CI = 0.79–1.25), time from ICU admission to death (OR = 0.94; 95% CI = 0.84–1.05), and time from ventilation to death (OR = 0.94; 95% CI = 0.84–1.06). These reductions were not statistically significant (Table 8).

**TABLE 8:** The effect of hospital choice on different patient outcomes.

Predictor	Estimate	SE	Odds ratio	95% confidence interval		*P*‐value
				Lower	Upper	
Hospital time (Days)	−0.08	0.05	0.93	0.83	1.03	0.161
Time to ICU (Days)	−0.05	0.17	0.95	0.68	1.32	0.749
Time to vent (Days)	−0.01	0.12	0.99	0.79	1.25	0.944
ICU to death time (Days)	−0.07	0.06	0.94	0.84	1.05	0.263
Ventilation to death (Days)	−0.06	0.06	0.94	0.84	1.06	0.308

*Note*: Effect of hospital choice on patients' outcomes (Hospital B compared with Hospital A as a reference).

## 4 DISCUSSION

In our study, we compared a hospital in the Saudi Western Province (Medina) to a hospital in the Saudi Eastern Province (Dammam). The COVID‐related mortalities during the observed duration were similar between the two hospitals/provinces. This is consistent with the Saudi official records where the total cases in the Eastern Province were 82,072, with overall deaths of 557 (mortality rate of 0.68%).^24^ In the same context, the total cases in Medina were 23,272, with overall deaths of 132 (mortality rate of 0.57%).^24^ Overall, Saudi Arabia's case fatality rate is also among the lowest fatality rates in the world, ranging from 0% to 28.9%.^25^ According to our results, it is consistent in different regions of Saudi Arabia, which supports that the quality of healthcare is relatively homogenous and of adequate quality.

Our results showed a relatively consistent presentation of clinical symptoms/signs among the included patients in comparing the two hospitals. Nevertheless, there were some differences in the initial presentation, including the initial body temperature, initial heart rate, breathing rhythms, breathing quality, and added sounds. Many of the previously reported MERS‐CoV^26^ and SARS‐CoV^27^ patients also showed similar comorbidities, which predisposed to increasing the risk of infection with MERS‐CoV and increasing the case fatality rates.^28^ Regarding clinical presentation, the predominant presentations among COVID‐19 patients were low‐grade high fever (mean temperature 37.7) and cough, which seems to be consistent with the initial reports from different countries.^3^, ^12^, ^29^, ^30^, ^31^ According to our results, treatments used were homogenous among the two hospitals, except for heparin use. Others have reported that many COVID‐19 patients suffer from a hypercoagulability state.^32^, ^33^

To our knowledge, this is the first study to compare COVID‐patients in two Saudi hospitals in two different provinces. However, the study has some limitations. The relatively small number of included patients may affect the magnitude of differences and the statistical significance. Moreover, some patients' data were missing, which may also affect our results.

## 5 CONCLUSION

Throughout the COVID‐19 outbreak in Saudi Arabia, the Kingdom has maintained a robust healthcare system and minimized case fatalities. we found a relatively consistent presentation of clinical symptoms/signs among the included patients in comparing the two hospitals. The majority of COVID‐19 deaths occur mainly among men, which is consistent with global reports of COVID‐19 fatalities and Saudi Arabia's COVID‐19 case distribution. Patients who died typically presented with more severe illnesses and were more likely to have underlying health conditions. Migrant workers may be at increased risk due to poorer baseline health and reluctance to seek care. This highlights the importance of cross‐cultural outreach to prevent deaths. Health education efforts should be multilingual and accommodate all literacy levels. Following further validation, heparin should be considered on a wider scale in the Western region (Medina) since we noticed a major gap in usage.

## AUTHOR CONTRIBUTIONS

**Fatimah Saeed AlGhawi:** Conceptualization; formal analysis; investigation; methodology; project administration; validation; writing—original draft; writing—review and editing. **Abdullah M. Assiri:** Conceptualization; formal analysis; investigation; methodology; project administration; supervision; validation; writing—review and editing. **Sami AlMudarra:** Conceptualization; investigation; methodology; project administration; supervision; validation; writing—review and editing.

## CONFLICT OF INTEREST STATEMENT

The authors report no conflict of interest.

## ETHICS STATEMENT

The study was approved by the institutional review boards at King Fahad Medical City, Riyadh, Saudi Arabia.

### PEER REVIEW

The peer review history for this article is available at https://www.webofscience.com/api/gateway/wos/peer-review/10.1111/irv.13127.

## Data Availability

The additional data that support the findings of this study are available from the corresponding author upon reasonable request.
